# Supplementary material for: Aqueous Extract of Sea Squirt (Halocynthia roretzi) with Potent Activity against Human Cancer Cells Acts Synergistically with Doxorubicin
Source: Mar Drugs. 2022 Apr 23;20(5):284. doi: 10.3390/md20050284 (PMC9143001; doi:10.3390/md20050284)
Supplement: Supplementary file 1 [file marinedrugs-20-00284-s001.zip › marinedrugs-1623618-supplementary.pdf]

SUPPLEMENTARY MATERIALS

Aqueous extract of Sea Squirt (*Halocynthia roretzi*) with Potent Activity against Human Cancer Cells Acts Synergistically with Doxorubicin

Yuting Zhu[1,2], Shanhao Han[1,2], Jianhui Li[1,2], Hongwei Gao[3,*] and Bo Dong[1,2,4,*]

1 Sars-Fang Centre, MoE Key Laboratory of Marine Genetics and Breeding, College of Marine Life Sciences, Ocean University of China, Qingdao 266003; yutingzhu19@hotmail.com (Y.Z.); hanshanhao@stu.ouc.edu.cn (S.H.); lijianhui@stu.ouc.edu.cn (J.L.); bodong@ouc.edu.cn (B.D.)

2 Laboratory for Marine Biology and Biotechnology, Qingdao National Laboratory for Marine Science and Technology, Qingdao 266237, China;

3 Technology Center of Qingdao Customs, Qingdao 266002, China，ghw75@126.com (H.G.)

4 Institute of Evolution & Marine Biodiversity, Ocean University of China, Qingdao 266003, China.

* Correspondence: ghw75@126.com (H.G.); bodong@ouc.edu.cn (B.D.); Tel.: +86-0532-82032732

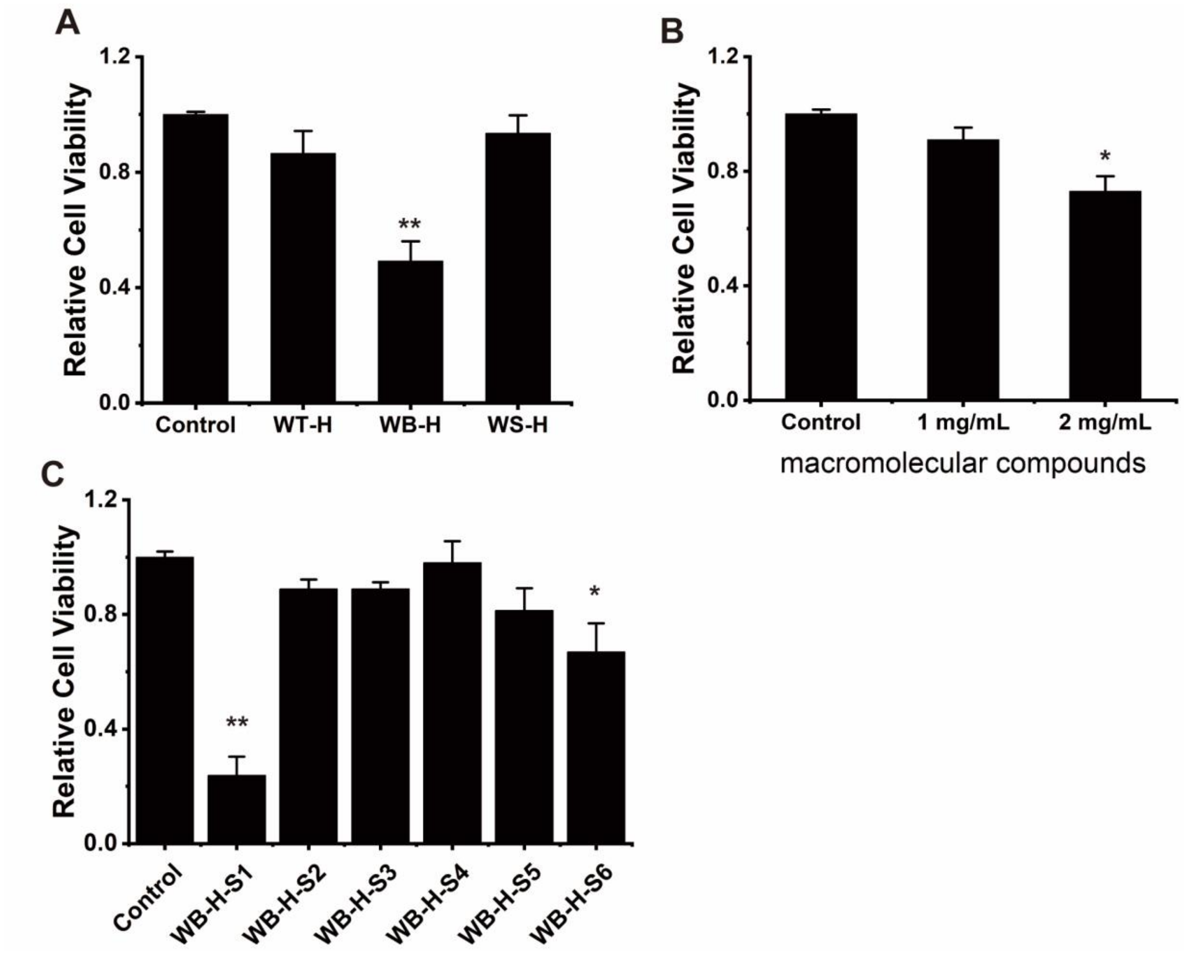

Figure S1. (A) HepG 2 cells were treated with aqueous extracts from tunic (WT-H), body tissue (WB-H), and stolon (WS-H) from *H. roretzi* at concentration of 100 μL/mL for 24 h. Relative cell viability was measured by MTT assay. (B) HepG 2 cells were treated with macromolecule part of WB-H at concentration of 1 mg/mL and 2 mg/mL or with same volume of distilled water for 48 h. (C) Small molecular part of WB-H was divided into six components. HepG 2 cells were treated with six parts at concentration of 400 μg/mL or the same volume of methanol (Control) for 48 h. Data are mean ± SEM, n = 3, *$p < 0.05$, **$p < 0.01$.

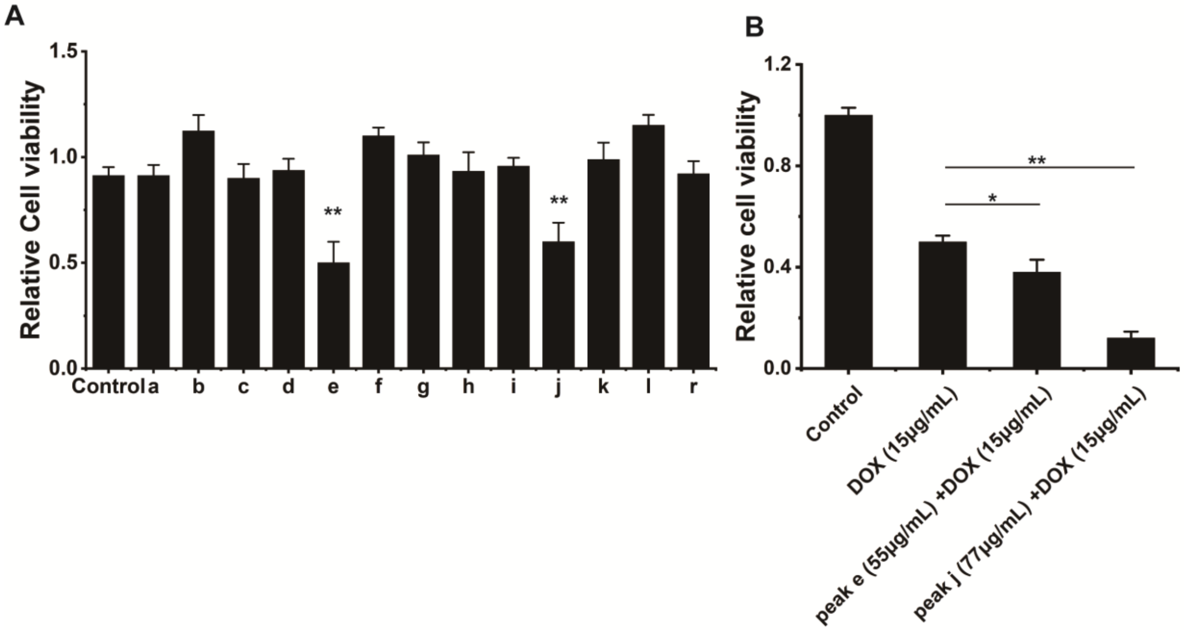

Figure S2. (A) HepG 2 cells were treated with compounds from peaks a-l and the rest of the eluting portion (r) at concentration of 50 μg/mL or methanol (Control) for 48 h. Relative cell viability was measured by MTT assay. (B) HepG 2 cells were exposed to doxorubicin at the concentrations of 15 μg/mL for 48 h in the presence or absence of peak e or peak j at the concentrations of 55 μg/mL and 77 μg/mL, respectively. Relative cell viability was measured by MTT assay. Data are mean ± SEM, n = 3, *$p < 0.05$, **$p < 0.01$.

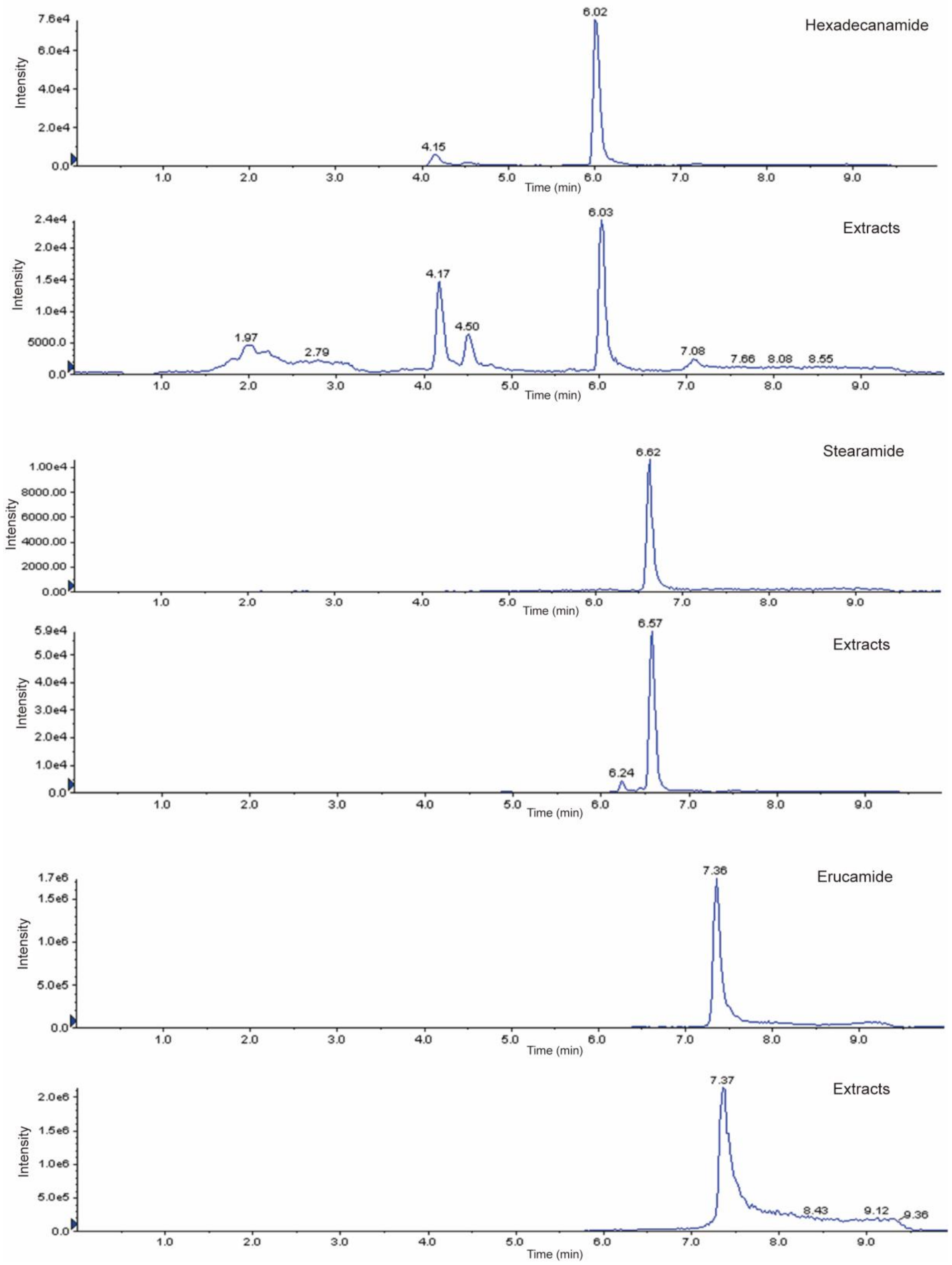

Figure S3. Ion chromatograms of fatty acid amides from *H. roretzi* extracts and standards.

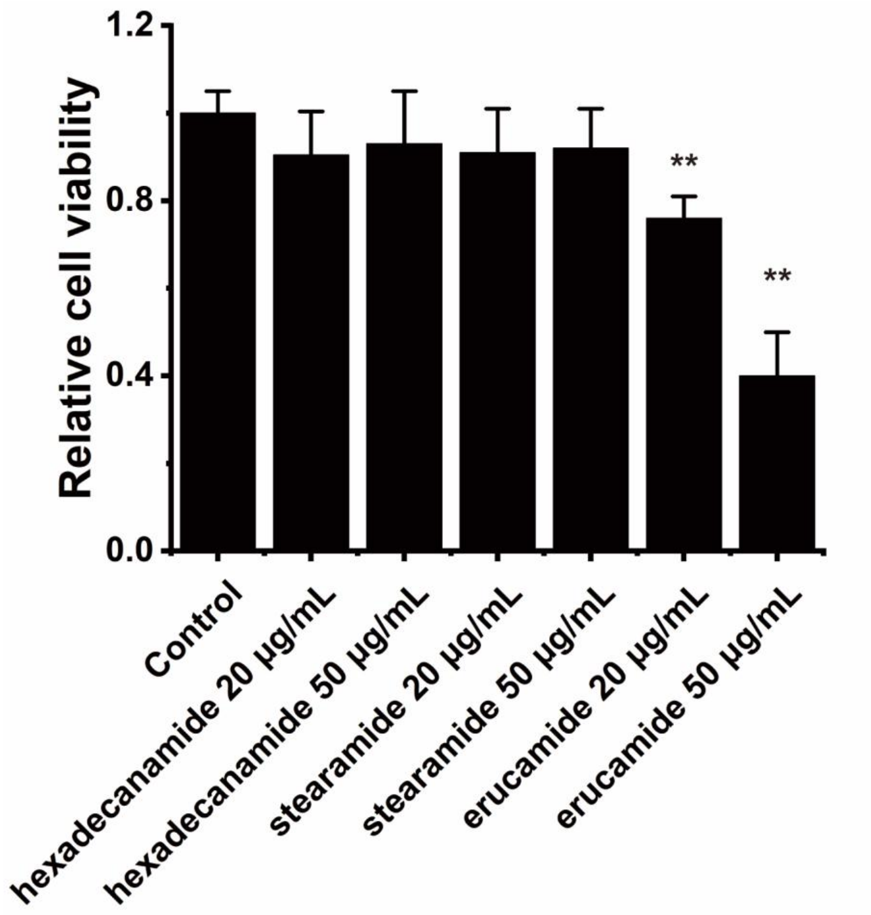

Figure S4. HepG 2 cells were treated with hexadecanamide, stearamide, and erucamide, respectively at concentration of 20 μg/mL and 50 μg/mL or methanol (Control) for 48 h. Relative cell viability was measured by MTT assay. Data are mean ± SEM, n = 3, **$p < 0.01$.
